# Evaluating the health effects of place-based slum upgrading physical environment interventions: A systematic review (2012–2018)☆

**DOI:** 10.1016/j.socscimed.2020.113102

**Published:** 2020-06-15

**Authors:** Rosie Mae Henson, Ana Ortigoza, Kevin Martinez-Folgar, Fernando Baeza, Waleska Caiaffa, Alejandra Vives Vergara, Ana V. Diez Roux, Gina Lovasi

**Affiliations:** a Urban Health Collaborative, Dornsife School of Public Health, Drexel University, 3600 Market Street, 7th Floor, Philadelphia, PA, USA, 19104; b Instituto de Nutrición de Centroamérica y Panamá (INCAP), Guatemala; c Departamento de Salud Pública, Escuela de Medicina, Pontificia Universidad Católica de Chile, Chile; d Observatório de Saúde Urbana de Belo Horizonte, Universidade Federal de Minas Gerais, Brazil; e Centro de Desarrollo Urbano Sustentable (CEDEUS), Pontificia Universidad Católica de Chile, Chile

**Keywords:** Slum upgrading, Urban upgrading, Health impact, Urban environment, Built environment, Low-to-middle income countries

## Abstract

Rapid urbanization in low- and middle-income countries (LMIC) is associated with increasing population living in informal settlements. Inadequate infrastructure and disenfranchisement in settlements can create environments hazardous to health. Placed-based physical environment upgrading interventions have potential to improve environmental and economic conditions linked to health outcomes. Summarizing and assessing evidence of the impact of prior interventions is critical to motivating and selecting the most effective upgrading strategies moving forward. Scientific and grey literature were systematically reviewed to identify evaluations of physical environment slum upgrading interventions in LMICs published between 2012 and 2018. Thirteen evaluations that fulfilled inclusion criteria were reviewed. Quality of evaluations was assessed using an adapted Effective Public Health Practice Project Quality Assessment Tool for Quantitative Studies. Findings were then pooled with those published prior to 2012. Narrative analysis was performed. Of thirteen evaluations, eight used a longitudinal study design (“primary evaluations”). All primary evaluations were based in Latin America and included two housing, two transportation, and four comprehensive intervention evaluations. Three supporting evaluations assessed housing interventions in Argentina and South Africa; two assessed a comprehensive intervention in India. Effects by intervention-type included improvements in quality of life and communicable diseases after housing interventions, possible improvements in safety after transportation and comprehensive interventions, and possible non-statistically significant effects on social capital after comprehensive interventions. Effects due to interventions may vary by regional context and intervention scope. Limited strong evidence and the diffuse nature of comprehensive interventions suggests a need for attention to measurement of intervention exposure and analytic approaches to account for confounding and selection bias in evaluation. In addition to health improvements, evaluators should consider unintended health consequences and environmental impact. Understanding and isolating the effects of place-based interventions can inform necessary policy decisions to address inadequate living conditions as rapid urban growth continues across the globe.

## 1 Introduction

The link between urban environments and health is well-established. Abundant research largely from high income countries has documented associations of neighborhood built and social environments including measures of walkability, physical activity infrastructure, healthy food availability, green space, traffic and air pollution and violence with health outcomes (Gascon et al., 2016; Khreis et al., 2017; MacMillan et al., 2018; Mayne et al., 2015; Mazumdar et al., 2017; Wang et al., 2016). Similarly, research on housing and health indicates a strong link between housing conditions and physical and mental health (Bassuk et al., 2015; Haines et al., 2013; Sharpe et al., 2018; Thomson et al., 2009; Tusting et al., 2015). Research in low and middle income countries (LMICs), although more limited, also indicates links between urban physical and social environments and health (Agampodi et al., 2015; Barbosa Filho et al., 2016; Gaihre et al., 2016; Giles-Corti et al., 2016; Sarmiento et al., 2015).

Rapid urbanization has shifted the proportion of the world’s population living in cities and towns from 5% to 50% over the past two centuries (Ezeh et al., 2017). By 2030, it is estimated that 5 billion of the world’s projected 8.1 billion people will live in urban areas (United Nations, n. d.). The majority of this massive urban growth has occurred in LMICs (United Nations, n. d.), where urbanization without adequate planning or infrastructure capacity has contributed to the development of informal settlements or slums. The term slum has been critiqued as pejorative because it can elicit negative stereotypes about the individuals who live in slums (Gilbert, 2007). However, it is routinely used by international development agencies to characterize areas and living conditions lacking basic shelter needs (UN-Habitat, n. d.). We use it here to refer to heterogeneous areas lacking basic shelter needs, as well as other physical, social, economic, and legal disenfranchisement resulting from structural exclusion.

Of the 5 billion projected to live in urban areas in 2030, 2 billion will live in slums, mainly in Africa and Asia (United Nations, n. d.). People who live in slums often face multiple disadvantages, including poverty, income inequality, insecurity of tenure and absence of other rights, as well as hazardous conditions in the physical environment (Ezeh et al., 2017). Physical environments can be hazardous due to cramped living spaces, dangerous topography (e.g. ravines, flood plains), precarious or loose fitting building materials, insufficient insulation from extreme temperatures, contaminated water and sanitation systems, lack of connection to city utilities and waste disposal, and little open space for play, relaxation and social bonding (Ezeh et al., 2017). The social environments of slums are sometimes characterized by high levels of violence and lack of opportunities for education and jobs (Corburn and Sverdlik, 2018).

Rapid urbanization, especially in LMICs, and the associated increase in the number of persons living in informal settlements and slums have highlighted the importance of interventions aimed at improving living conditions in these neighborhoods. Improving the health, well-being, and environmental sustainability of these settlements is critical to achieving Sustainable Development Goal 11 (United Nations, n. d.). Slum upgrading is an umbrella term that refers to the process of improving environmental, social, economic, and legal conditions in slums through interventions related to land tenure, housing, infrastructure, employment, education, health services and social inclusion (UNHabitat). Here we focus on place-based physical environment upgrading, including housing improvements, connections to utilities, road paving, development of public spaces, and implementation of transit options with or without the integration of broader upgrading interventions (Turley et al., 2013). Such place-based upgrading has the potential to foster long-term improvement in environmental and economic conditions linked to health outcomes with less disruption to the social environment (Lilford et al., 2017).

Summarizing evidence of the impact of prior interventions is critical to motivating and selecting the most effective strategies going forward. The quantity and quality of evidence for the effect of these interventions on health, however, remains limited. Assessing past and current interventions can guide cities and organizations seeking to implement place-based slum upgrading interventions by highlighting the types of interventions undertaken and the pathways through which population health is most likely to be affected. In addition, the strengths and limitations of previous evaluations can guide future evaluations.

A review published in 2013 summarized the health impact of place-based physical environment slum upgrading interventions. This review found a small number of evaluations across the period of 1986–2012 and concluded that heterogeneity and evidence gaps prevented strong conclusions regarding the effect of physical environment upgrading on health and well-being (Turley et al., 2013). In this manuscript, we update and expand on that work.

The purpose of this review is to: (1) describe the characteristics of physical environment slum upgrading interventions evaluated between 2012 and 2018; (2) assess the quality of evaluations conducted between 2012 and 2018; (3) synthesize health effects of physical environment slum upgrading interventions reported over the period 1986 to 2018; and (4) describe common challenges among these evaluations and recommendations for future research.

## 2 Methods

### 2.1 Search strategy

Literature was searched to identify evaluations published between 2012 and 2018. Databases were selected to reflect the cross-disciplinary nature of the topic, and cover health, social science, urban planning, and engineering. Databases searched included MEDLINE, Web of Science (Science Citation Index Expanded, Social Sciences Citation Index, Arts & Humanities Citation Index, and Emerging Sources Citation Index), Cochrane (Public Health Group and CENTRAL), ProQuest (Applied Social Sciences Index & Abstracts, GeoRef, and Sociological Abstracts), Avery Index to Architectural Periodicals, EI Compendex, and Greenfile. The search was conducted in January 2019. Databases were searched in all available languages. To provide an update with comparable coverage of relevant literature, search terms were replicated or adapted from the review by Turley et al. to match the query convention of each database (Supplementary Table 1). The search terms used reflected two domains: one related to the setting of slums and one to types of intervention. Search terms were used to identify the pool of all eligible studies of physical environment interventions in urban slum settings. Given the breadth of possible health outcomes assessed, search terms related to health were not included at the stage of identifying eligible studies. Inclusion of health outcomes was assessed at the abstract and full-text screening stages. Because some evaluations may not be reported in peer-reviewed literature, a comprehensive search of eighteen grey literature and development stakeholder websites was also conducted (Supplementary Table 2).

### 2.2 Selection of studies

#### 2.2.1 Inclusion criteria

To be considered as part of the update, studies had to satisfy inclusion criteria related to publication date, study design, intervention type, intervention setting, and health-relevant outcomes. Studies were included if they: (1) were published between 2012 and 2018 unless included in the prior review by Turley et al.; (2) used a randomized control trial (RCT), controlled before-after (CBA), interrupted time series (ITS), uncontrolled before-after (UBA), or controlled post-intervention (CPI) study design with at least 100 observations; (3) evaluated at least one intentional physical environment intervention, such as improvements to transportation infrastructure, water and sanitation, waste management, energy infrastructure, housing, or environmental hazard mitigation; (4) were place-based, set in an urban or peri-urban slum in a LMIC as assessed in 2018 fiscal year; and (5) assessed health outcomes or social, economic, and environmental outcomes stated as health-relevant by evaluation authors.

If not explicitly described as a slum setting, setting was determined using UN Habitat criteria for defining slums, following the same approach as Turley et al. For one evaluation, authors were contacted to determine study setting. As the main focus of the review is place-based physical environment interventions, studies that only assessed behavioral, educational, social or health service interventions without accompanying physical environment or infrastructure change in slums were excluded. For the same reasons, interventions to prevent slum formation or relocate slum dwellers outside of the slum territory were not considered for this review.

#### 2.2.2 Screening process

**Fig. 1:**
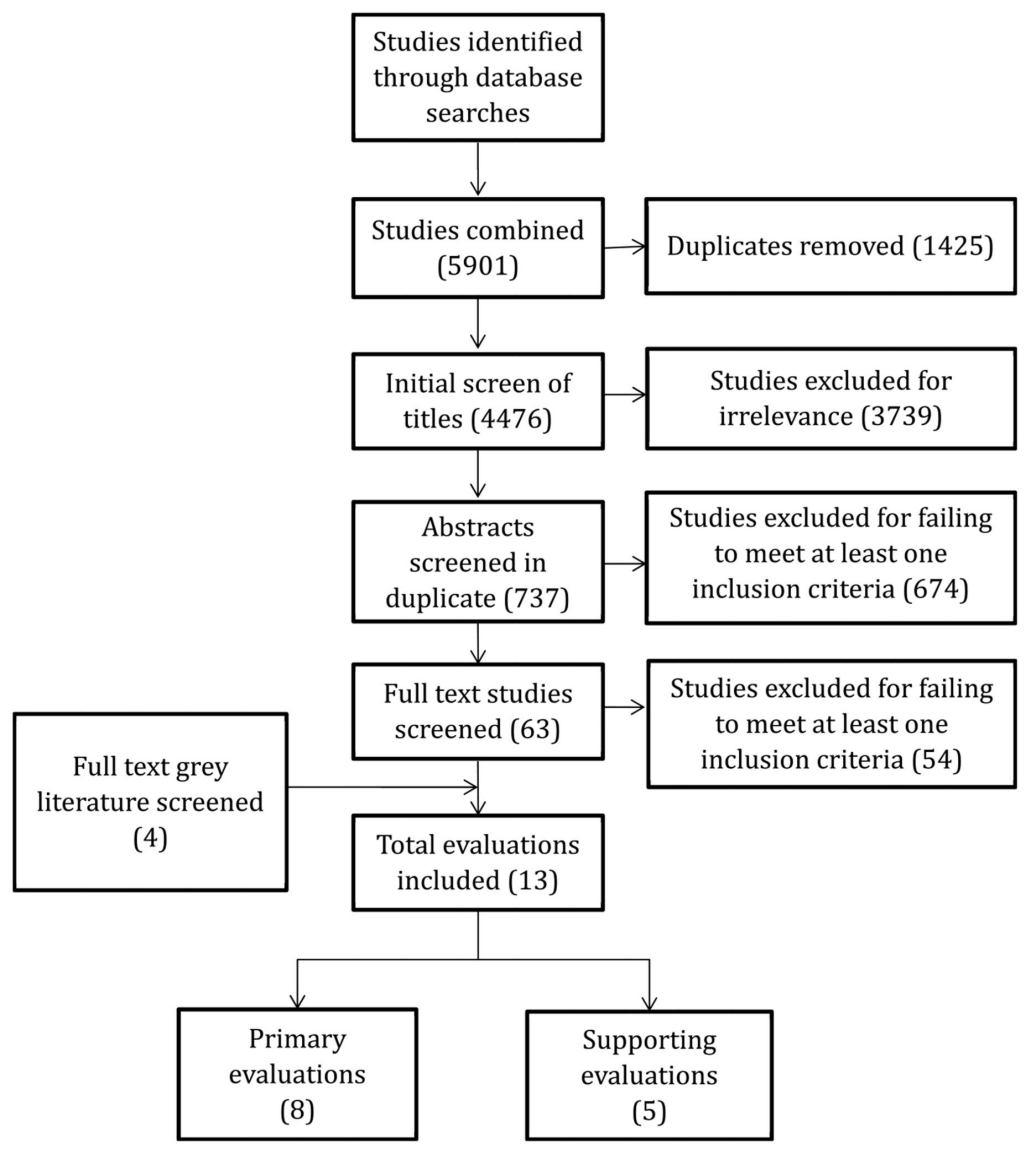
Sequence of the searching strategy.

The screening process is outlined in Fig. 1. All studies returned from the initial search were screened for duplicates by a single author (RMH). Unique studies were screened by title for relevance by a single author (RMH), with exclusions based on setting and intervention type. Abstracts of all remaining studies were screened for inclusion criteria in duplicate following training for agreement across three authors (RMH, AO, GL). The full text of potentially relevant articles (as determined by abstract screening) and all potentially relevant grey literature reports were screened for inclusion criteria in duplicate by two authors (RMH, AO). Any disagreement about inclusion was resolved by discussion between screening authors. Total studies included in the review were categorized as “primary evaluations” if they used a more rigorous RCT, CBA, or ITS study design and “supporting evaluations” if they used a less rigorous UBA or CPI study design with limited ability for causal inference.

### 2.3 Data Extraction and synthesis

Information from the screened studies was extracted in duplicate by three authors (RMH, AO, KMF). Information was extracted from both primary and supporting evaluations due to the small overall number of included evaluations. Although causal inference is not possible from supporting studies, they can still indicate associations between inter-ventions and outcomes. Including these studies allows for assessing consistency with primary evaluations and describing the available evidence for interventions, settings, and health outcomes not available from primary evaluations. Information collected from evaluations included study design, unit of analysis, geographic region, main intervention type assessed, main outcomes of interest, covariates considered in analysis, and study findings. Information from evaluations included in the review by Turley et al. was extracted in duplicate by two authors (AO, RMH). Information collected from these evaluations included study design, geographic region, main intervention type assessed, main outcomes of interest, and study findings. Heterogeneity of interventions and outcome measures prevented pooling of studies for meta-analysis (Craig et al., 2012).

To provide an update to Turley et al. and as recommended for systematic reviews of heterogeneous evidence (Siddaway et al., 2019; Snilstveit et al., 2012), narrative analysis of evaluation characteristics and findings was performed for evaluations published between 2012 and 2018. This analysis was stratified by primary and supporting evaluations due to study design and quality differences previously described. Narrative analysis of intervention effects on outcomes was then performed, pooling data from all evaluations between 1986 and 2018 to reduce limitations due to heterogeneity.

### 2.4 Quality assessment

Quality of evaluations published between 2012 and 2018 was assessed using an adapted version of the Effective Public Health Practice Project Quality Assessment Tool for Quantitative Studies (Effective Public Health Practice Project, 1998) (Supplementary Table 3). This tool was chosen because of its documented and accepted validity and reliability as a quality appraisal tool (Thomas et al., 2004) and its ability to accommodate multiple study designs, including both randomized trials and observational studies. Originally designed for clinical settings, the tool was adapted to account for the less controlled nature of place-based physical environment interventions. This included adding scoring guidelines for the intervention integrity domain due to greater inconsistency in intervention exposure in non-clinical settings, and for the analysis domain due to the frequent inability to control intervention allocation through study design and the relatively greater importance of statistical adjustment. The adapted tool was piloted by two authors (RM, AO). For piloting, Cohen’s kappa statistic for interrater reliability across domains was between 0.43 and 1.0 (moderate to almost perfect agreement), and disagreements were resolved by discussion. Domains assessed in the quality assessment included selection bias, study design, intervention integrity, blinded outcome assessment, data collection methods, withdrawals and drop-outs, confounders, and analyses. Raters were trained on scoring guidelines. Quality assessment for evaluations was completed in duplicate by three authors (RMH, AO, KMF). Any remaining disagreement in domain quality scores was resolved by discussion.

## 3 Results

### 3.1 Search results

The database searches returned 4476 unique articles (Fig. 1). The titles of these articles were screened for relevance, and 3739 were excluded. Abstracts of the remaining 737 articles were screened in duplicate, resulting in 674 studies excluded for failing to meet at least one inclusion criteria. The full text of sixty-three articles from database searches and four articles from grey literature and snowball searches were screened. Thirteen articles met the criteria for inclusion based on full text. Eight of these thirteen were classified as “primary” evaluations because they utilized randomized controlled trial, controlled beforeafter, or interrupted time series designs. Five were classified as “supporting” evaluations because they utilized uncontrolled before-after or controlled post-intervention designs.

### 3.2 Description of evaluations included in the update: 2012–2018

Table 1 provides an overview of all physical environment slum upgrading evaluation characteristics. Supplementary Table 4 includes a detailed summary of primary and supporting evaluations included in the update.

#### 3.2.1 Primary evaluations

##### 3.2.1.1 Study Ddesign

The primary evaluations included eight articles on five interventions in five Latin American countries. Six articles assessing three interventions used a randomized control trial design, and two articles evaluating two interventions used a controlled beforeafter design (Table 1, panel A).

##### 3.2.1.2 Interventions

###### 3.2.1.2.1 Single interventions

Two articles evaluated the TECHO program, a stand-alone housing intervention implemented in several Latin American countries by a non-governmental organization (Galiani et al., 2018; Galiani et al., 2017). TECHO provided prefabricated and movable 18 m^2^ housing units with limited amenities (i.e. no bathroom, kitchen, plumbing, water hook-ups or gas connection).

Two articles evaluated two transportation infrastructure interventions: the Metrocable public transportation system (Cerdá et al., 2012) and first-time street paving (Gonzalez-Navarro and Quintana-Domeque, 2016). The Metrocable is a cable-propelled public transportation system that connects Medellin city center to neighborhoods in the mountainous periphery, and included accessibility considerations like transit police, footpaths, and lighting. First-time street paving was a one-time intervention that involved asphalting of residential non-arterial streets in the Mexican city of Aracuyan with no other physical improvement in the nearby areas.

###### 3.2.1.2.2 Comprehensive interventions

Four articles evaluated two comprehensive neighborhood upgrading programs: Mexico’s Programa Hábitat (McIntosh et al., 2018; Ordóñez Barba et al., 2013; Ordóñez Barba and Ruiz Ochoa, 2015) and Brazil’s PAC-Vila Viva program (Friche et al., 2015). Hábitat was a federal program primarily oriented to building physical infrastructure, such as roads, water, sewerage, lighting, and sidewalks in high poverty urban areas in Mexico. The program also included the creation of community centers for job training and childcare centers. PAC Vila Viva was a local program in Belo Horizonte that included physical as well as legal, social and organizational reforms in slum areas. Physical environment upgrades included improvements to roadways, public lighting, alleys and green areas, installation of public equipment and areas for cultural and social activities, construction of parks, and slope stabilization. Sanitation upgrades included improvements to drainage, running water supply, sewage systems, and urban cleaning. Housing upgrades included construction of new housing units and land titling.

##### 3.2.1.3 Outcome measures

The majority of primary evaluations (five articles on four interventions) included some measure of personal or neighborhood safety as an outcome (Table 1, panel A). Measures included perceived security, feeling safe walking at night, physical assault, teen behavior, feeling safety limits activities, perceived violence, and annual neighborhood homicide rate. A few of the primary evaluations (three articles on three interventions) reported on communicable disease outcomes. Measures included self-reported or caregiver-reported child respiratory and child diarrhea episodes in the past month, fungus/parasite skin infections, and diarrhea and skin infection episodes within three months. Two evaluations of one intervention reported on quality of life as an outcome, one of which assessed the persistence of quality of life changes over time. Two evaluations of one intervention reported on social capital using an index of social capital. One evaluation assessed general physical health with a measure of sickness in the previous month. One evaluation included cause-specific mortality rates as an outcome, specifically mortality from infectious diseases, non-communicable diseases, and external causes (Table 1, panel A). No primary evaluations included measures for maternal/perinatal outcomes, nutritional deficiencies, general mental health, injuries, or non-communicable diseases.

##### 3.2.1.4 Findings: physical environment upgrading effects by outcome category

The effects of interventions on safety outcomes were mixed in primary evaluations. In the single TECHO housing evaluation, perceptions of security significantly increased in El Salvador, but not in Mexico or Uruguay, likely due to baseline differences. A single transportation intervention evaluation of street paving in Mexico found no effect on feelings of safety while walking, while a single transportation intervention evaluation of Colombia’s cable-car system reduced perceived violence and the annual neighborhood homicide rate. The comprehensive Programa Hábitat in Mexico reduced physical assault and teen misbehavior, and improved perceptions of whether safety limited activities (Table 2, panel A). Communicable disease outcomes also had mixed findings. The single TECHO housing intervention found reductions in child respiratory and diarrhea episodes in the last month, and the comprehensive Programa Hábitat found reductions in diarrhea and skin infection episodes in the past three months. Single first-time street asphalting, however, found no significant effects on fungus and parasite skin infections (Table 2, panel A). The remaining outcome categories were reported on by only one intervention in primary evaluations. The single TECHO housing intervention improved quality of life, with one evaluation reporting diminishing improvements in quality of life over time. The comprehensive Programa Hábitat had no significant effects on social capital. The single street paving transportation intervention in Mexico had no significant effects on sickness in the past month. Evaluation of the comprehensive PAC Vila Viva program found reductions in mortality rates, but no significance testing was conducted (Table 2, panel A).

#### 3.2.2 Supporting evaluations

##### 3.2.2.1 Study Ddesign

Supporting evaluations included five articles of four interventions in Argentina, South Africa, and India. One article on one intervention used an uncontrolled before-after design, and four articles on three interventions used a controlled post-intervention design (Table 1, panel B).

##### 3.2.2.2 Interventions

###### 3.2.2.2.1 Single interventions

One article evaluated the TECHO program, a stand-alone housing intervention described in the previous section (Simonelli et al., 2013). Two articles evaluated the South African housing subsidy programs: one evaluation described the housing subsidy program generally, in which low-income residents receive subsidies for housing construction and upgrading (Marais and Cloete, 2014), and one evaluation described local implementation through the People’s Housing Process, in which the local community guides implementation of subsidy-based housing upgrades and construction (Shortt and Hammett, 2013).

###### 3.2.2.2.2 Comprehensive interventions

Only one comprehensive intervention was evaluated among the supporting evaluations. Two articles evaluated the Slum Networking Program, a community-driven physical environment upgrading intervention in regions of India (Parikh et al., 2012; Parikh et al., 2015). The program provided integrated household-level infrastructure, including water, sanitation, and electricity.

##### 3.2.2.3 Outcome measures

In the majority of supporting evaluations (three articles; three interventions), general physical health was assessed (Table 1, panel B). Measures included physical health, children’s nutritional status, adult asthma, illness and injury, selfrated health, disease rate, monthly medical expenditure, and monthly workdays lost due to illness. Two articles of two interventions assessed communicable disease outcomes, including measures of infectious disease, tuberculosis, and skin disorders. Two articles on two interventions assessed quality of life using a quality of life measure and a measure of health as a higher order social aspiration (relative to “lower order” basic physical needs). Two articles of two interventions included social capital outcomes. Measures included social relationships and community belonging. Two articles on two interventions assessed general mental health, including measures of psychological wellbeing, sleep quality, and mental illness. The remaining outcome categories were reported on by only one intervention among supporting evaluations. One evaluation assessed injuries (self-reported injury), nutritional deficiencies (children’s nutritional status), non-communicable diseases (adult asthma), and mortality (infant and child mortality) (Table 1, panel B). Unlike the primary evaluations, in which safety measures represented the most commonly assessed outcome, supporting evaluations included no safety outcomes. Maternal/perinatal outcomes were also not reported in supporting evaluations.

##### 3.2.2.4 Findings: physical environment upgrading effects by outcome category

The effects of interventions on general physical health outcomes were mixed within supporting evaluations (Table 2, panel B). The single TECHO housing intervention reported an improvement in physical health one month after housing upgrade was completed, but found no significant difference after six months. The single South African subsidized housing intervention found no significant effect on self-rated health. children’s nutritional status, nor adult asthma. It was, however, associated with lower illness and injuries. The comprehensive Slum Networking program was associated with lower disease rates, monthly medical expenditures, and monthly workdays lost due to illness. In two articles, the single South African subsidized housing intervention found no significant associations on communicable disease outcomes, including infectious disease symptoms, tuberculosis, and skin disorders. Both supporting evaluations reported improvements in quality of life. The single housing TECHO intervention was associated with better quality of life assessments and the comprehensive Slum Networking intervention was associated with better quality of life as described by the authors’ framework for the satisfaction of needs (Table 2, panel B). The effects of interventions on social capital were also mixed. The single housing TECHO intervention had no significant associations with social relationships, while the single South African subsidized housing intervention was associated with improved community belonging. Interventions, in general, were reported as being associated with better mental health outcomes. Specifically, mental illness was lower while sleep quality and psychological wellbeing were higher after single housing interventions of the People’s Housing Project and TECHO. The effects on psychological wellbeing, however, were no longer significant after six months. The one evaluation assessing injuries, nutritional deficiencies, noncommunicable diseases, and mortality found no significant associations after single housing intervention on reported injury, children’s nutritional status, adult asthma, or infant and child mortality rates, respectively. (Table 2, panel B).

### 3.3 Description of all evaluations: 1986–2018

#### 3.3.1 Evaluation characteristics

The pooled intervention and outcome data from this update and the Turley review increased the number of evaluations to twenty-five, of which twelve qualified as primary evaluations and thirteen as supporting evaluations. Nineteen interventions were evaluated, of which eleven were comprehensive interventions, five were single housing interventions, two were single transportation interventions, and one was a single sanitation intervention. Table 1 includes a summary of evaluation characteristics across both reviews.

#### 3.3.2 Findings: Intervention effects by outcome category

Table 2 provides a synthesis of health effects by each intervention type.

#### 3.3.3 Single housing interventions

The findings of single housing interventions on quality of life were the most consistent with positive effects for six measures of quality of life across both primary and supporting studies (Table 2). All results were assessed within Latin America, and most results came from evaluations of TECHO in four Latin American countries. Single housing interventions also had positive effects on communicable diseases, finding positive effects on two measures in primary studies and one measure in a supporting study all within Latin America. Supporting studies also reported non-significant effects of single housing interventions on communicable disease outcomes, particularly for the South African Housing subsidy (Table 2). No general physical or mental health outcomes were included in primary evaluations of single housing interventions. Supporting evaluations of single housing interventions reported mixed positive and non-significant effects on these outcomes for interventions in Latin America and Africa.

#### 3.3.4 Single transportation interventions

The two evaluations of single transportation interventions within Latin America found mixed results on safety and violence outcomes. Evaluation of the Metrocable found positive effects on two measures of safety, while street paving found no significant effects on two different measures of safety (Table 2).

#### 3.3.5 Comprehensive interventions

Evaluations of comprehensive interventions presented the most mixed results across outcome categories. Pooled, evaluations of comprehensive interventions included the most communicable disease outcome measures (Table 2). Among primary studies, non-significant effects were found across four measures and a positive effect was found on one measure. In supporting studies, most measures of communicable disease were untested, and a positive effect was found on one measure. Overall, the evidence suggests no significant effects of comprehensive interventions on communicable diseases. However, geographic variation of effects on communicable diseases was reported: positive effects were found for evaluations within Southeast Asian and non-significant effects were found in evaluations within Latin America. Effects of comprehensive interventions on safety outcomes were mixed (Table 2). Of the two comprehensive interventions evaluated for changes in safety outcomes, positive effects were found for two out of three measures for a national program in Mexico while no significant effects were found on the third measure and in an evaluation in Rio de Janeiro. The effects of comprehensive interventions on social capital were largely non-significant (Table 2). Two evaluations of one intervention assessed social capital, and found no effect on social capital. A supporting study using a different measure of social capital found a positive effect. General physical health was not assessed by any primary evaluations of comprehensive interventions. In supporting evaluations, positive effects were found on three measures of general physical health and were untested on four measures (Table 2).

### 3.4 Summary of quality assessment of evaluations included in the update

**Fig. 2:**
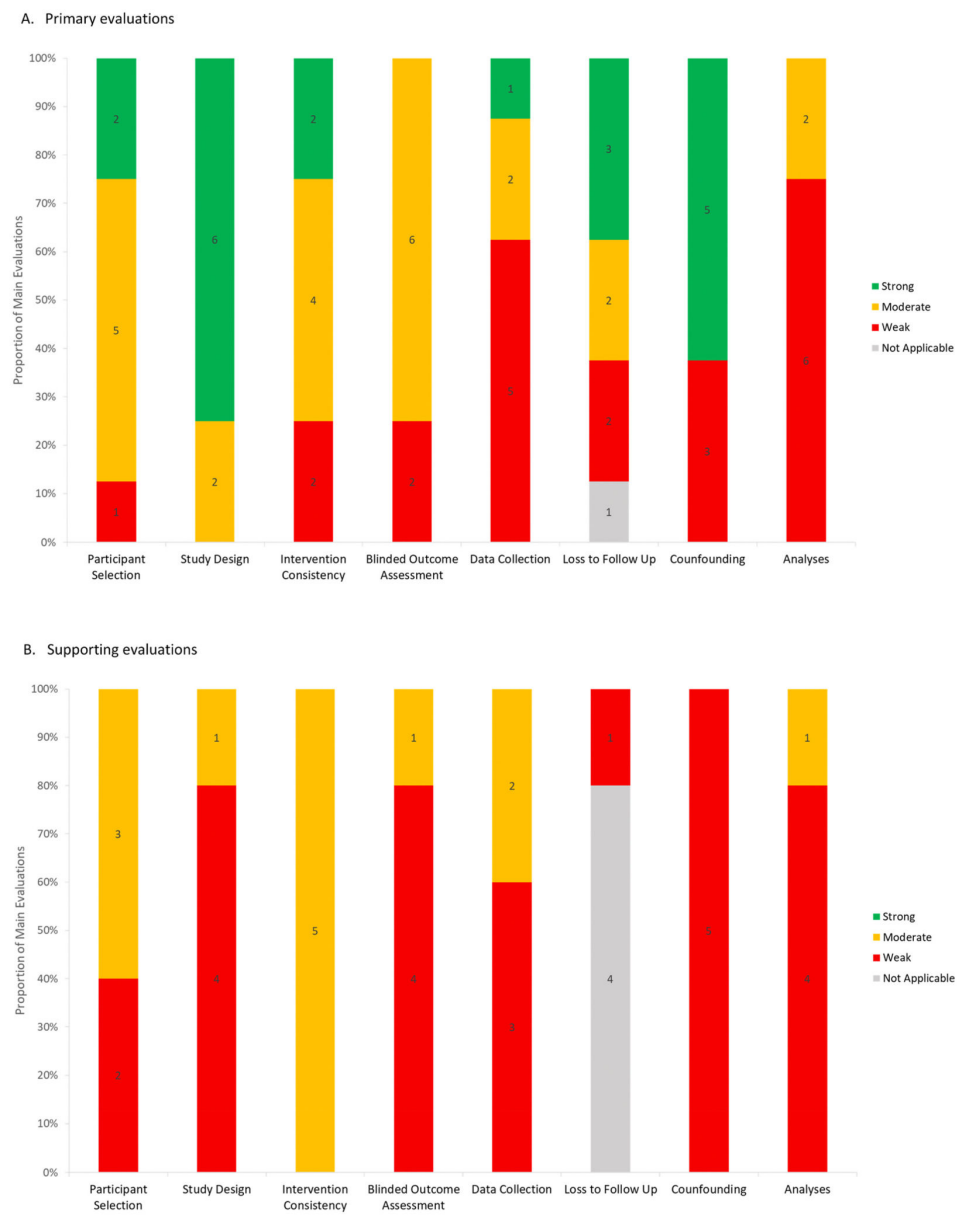
Quality assessment of evaluations published between 2012 and 2018 by domain.

Fig. 2 shows the distributions of the quality assessment results by domains of assessment for primary and supporting evaluations published between 2012 and 2018. Results of the quality assessment by article and domain are described in Supplementary Table 5.

#### 3.4.1 Primary evaluations

Quality of the evaluations varied within specific domains. In the study design domain, the majority of evaluations (6/8) scored strong and the remaining (2/8) scored moderate. In the confounding domain, the majority (5/8) scored strong. In the selection domain, the majority (5/8) scored moderate. In the intervention domain, half (4/8) scored moderate. In the blinded outcomes domain, the majority (6/8) scored moderate. In the loss to follow up domain, quality scores varied more widely with few scoring strong (3/8), moderate (2/8), and weak (2/8) and one scoring not applicable due to the use of trends in aggregated mortality data. In the analyses domain, the majority (6/8) scored weak and the remaining (2/8) scored moderate (Fig. 2, panel A).

#### 3.4.2 Supporting evaluations

In the study design domain, the majority of evaluations (4/5) scored weak and one scored moderate. In the confounding domain, all (5/5) scored weak. In the selection domain, approximately half (3/5) scored moderate and half (2/5) scored weak. In the intervention domain, all (5/5) scored moderate. In the blinded outcomes domain, the majority (4/5) scored weak and one scored moderate. In the loss to follow up domain, the majority (4/5) scored not applicable due to study design and one scored weak. In the analyses domain, the majority (4/5) scored weak and one scored moderate (Fig. 2, panel B).

## 4 Discussion

As urbanization and the associated number of individuals living in informal settlements increases, understanding the health effects of urban upgrading is important to inform the work of evaluators, researchers, and policymakers. We have updated and synthesized the best available evidence for the effects of physical upgrading interventions on health outcomes to meet this need. As is common in the systematic review of natural experiments (Craig et al., 2012), the breadth of intervention types and contexts, as well as study designs and study quality presented challenges in synthesizing the evidence from these interventions. This variation should be considered in the interpretation of these results and can provide insights and recommendations for future evaluations.

Health-related outcomes from primary and supporting evaluations included in the update (from the most to the least commonly assessed) were communicable disease, personal or neighborhood safety, social capital, quality of life, general mental health, general physical health, mortality, nutritional deficiencies, injuries, and non-communicable diseases. No evaluations reported negative (i.e. health harming) effects from the interventions; all effects reported were positive or null. This could reflect potential publication or reporting bias of positive health effects. Since Turley et al.‘s review, informal settlement upgrading evaluations have included an expanded set of health outcomes. For instance, in addition to communicable diseases, more recent evaluations have included measures of safety and violence (Cerdá et al., 2012; Galiani et al., 2017; Gonzalez-Navarro and Quintana-Domeque, 2016; McIntosh et al., 2018; Ordóñez Barba et al., 2013), quality of life (Galiani et al., 2017, 2018; Parikh et al., 2012; Simonelli et al., 2013), social capital (McIntosh et al., 2018; Ordóñez Barba and Ruiz Ochoa, 2015; Shortt and Hammett, 2013; Simonelli et al., 2013), and mental health (Shortt and Hammett, 2013; Simonelli et al., 2013). Although included outcomes have expanded, a lack of evidence for chronic health outcomes was noted in the previous review and persists. Chronic health outcomes, such as cardiovascular disease and diabetes, may not be included in evaluations of physical environment upgrading interventions hypothesized to affect these outcomes due to the long period of time usually necessary to observe changes in these outcomes. However, the inclusion of health-related behaviors, which could change more rapidly in response to an intervention, could be informative.

Some effects by intervention type emerged after pooling the results of evaluations included in the update with those from the previous review. The effects included improvements in quality of life and com-municable diseases after housing interventions, possible improvements in safety after transportation and comprehensive interventions, and possible non-statistically significant effects on social capital after comprehensive interventions. Effects due to interventions may vary, however, by regional context as evidenced by observed effects in some regional contexts but not others for housing and comprehensive interventions. The effects may also vary due to the scope of interventions. For example, the scope of the Metrocable transportation intervention (Cerdá et al., 2012) was much larger than street paving (Gonzalez-Navarro and Quintana-Domeque, 2016), which may explain detected improvements in safety after implementation of the Metrocable but not after street paving. In some cases, such as the effects on general physical and mental health outcomes in housing and comprehensive interventions, evidence was only available from supporting studies. In these cases, further investigation to determine intervention effects is needed by evaluations using more rigorous study designs. Overall, comprehensive interventions had largely non-statistically significant findings. It is possible that the multi-component nature of such interventions creates variations in intervention exposure that bias effects of the intervention toward to the null (Craig et al., 2012). Care is needed in drawing conclusions about the effects of specific types of physical environmental upgrading interventions given the heterogeneity and small number of included interventions.

In addition, some important types of interventions were not evaluated at all in the studies reviewed. For example, we found no studies that reported on evaluations of physical environment upgrades related solely to water supply and sanitation infrastructure, connection to electricity or other utilities, waste collection and drainage infrastructure, or green space. We also found no studies evaluating physical environment changes for improved environmental sustainability or climate resilience. Although some comprehensive interventions included upgrades to public spaces or parks, none of the evaluations focused specifically on the effects of intervention on these neighborhood spaces.

Quality assessment of evaluations included in the update highlighted areas where evaluations were rigorous and where improvement is needed. Primary evaluations scored the weakest in blinded outcome assessment, data collection, and analysis domains. Supporting studies scored the weakest in confounding, study design, blinded outcome assessment, and analysis domains. Supporting evaluation study designs allowed for the corroboration of results with primary evaluations, but the evidence from these studies cannot provide causal inference due to a lack of comparison data, which is a major limitation of these evaluations. Further, all supporting evaluations failed to adequately control for observable confounders, predominately adjusting only for individual sociodemographic characteristics like age and sex. A lack of use of validated and reliable measures in both primary and supporting studies led to considerable variation in outcome assessment, potentially introducing bias due to measurement error and limiting comparability of results. Further, mishandling or non-reporting of missing data in both primary and supporting studies introduced further opportunities for bias in evaluations.

Evaluators of urban interventions face many challenges in the design and conduct phases of an evaluation. The strengths and weaknesses noted during quality assessment in this review provide insights into recommendations for future evaluations (Table 3).

One domain for evaluators to consider is study design. While randomized controlled trial design is not feasible for some natural experiments, six experiments had the ability to compare change over time in intervention versus control communities. Such controlled before and after study designs minimize the potential for bias, provided control groups and intervention groups are balanced on relevant health determinants prior to the intervention. Such balance can be achieved in natural experiments through restriction or matching (Hannan, 2006). If such balance is not assured at the design phase, evaluators can later use techniques like propensity score weighting to estimate what the results would have been for balanced groups (Oakes and Johnson, 2006). While supportive studies are also noted, by lacking control groups or pre-intervention data such designs offer less flexibility at the time of analysis. Although less common, designs with random allocation to intervention or control conditions have been implemented, as illustrated by seven evaluations of three randomized controlled trials. The intervention itself is often outside of investigator control in such natural experiments, yet the evidence produced can be quite strong (Humphreys et al., 2016). Even when the organization implementing the intervention is committed to serving all eligible individuals or areas, it is often not feasible to implement the in-situ upgrading project for all eligible recipients simultaneously; randomly selecting recipient individuals or areas off a waiting list creates an opportunity for convincingly quantifying any health effects.

Planning for data collection in part relies on the study design. For designs with data collected over time, consistent use of the same validated measures on individuals will give the clearest picture of problems with balance between intervention and control groups before the intervention, allowing the effects of the intervention to be isolated. Loss to follow-up should be minimized by rigorous efforts to track and contact the same individuals for assessment. However, if loss to follow-up occurs, data collected to assess balance between groups can be used in sensitivity analysis to assess the extent of selection bias. Primary data collection can be efficiently complemented using secondary data sources (e.g. census data, hospital data, mortality records) that may contain data on outcomes, denominators, or health determinants for the intervention and control groups at multiple time points.

While study design and data collection plans may be more challenging and costly to refine, perhaps the greatest opportunity for more readily accessible improvements were noted in methodologic and analytic approaches. None of the studies met quality criteria for strong blinded outcome assessment, perhaps reflecting challenges due to the highly visible nature of physical environment interventions. However, at a minimum, quality of reporting can be enhanced by clearly stating to what extent individuals were aware of research questions or assessors were aware of participant’s intervention status. Also, secondary data that was collected for other purposes not related to the research question(s) of the evaluation may prove useful in assessing the degree of bias. Yet perhaps more strikingly, the quality criteria for strong analyses were also not met by any of the studies. Attention to potential confounding and missing data using techniques that have become common to observational research (such as adjustment, weighting, or imputation) remain important in the context of evaluation analyses (Field and Kremer, 2006).

Selecting the right outcome based on how the intervention might operate, measuring it in the right time frame, and using reliable and validated measures is critical to evaluation quality. For example it may be unrealistic to expect to see NCD mortality impacts of interventions within a relatively short timeframe or with few observed outcome events; lifestyle behaviors, risk factors, or biomarkers may be more sensitive to recent physical environment change. Evaluators should provide explicit description of hypothesized mechanisms connecting intervention and outcome through schemes or directed acyclic graphs that justify the research question and inclusion of covariates in analyses (Matthay and Glymour, 2020). Beyond health effects alone, future evaluations should also consider measuring the environmental impact of interventions together with their health effects, given the interconnectedness between environmental sustainability and human health (Etinay et al., 2018; Miller and Hutchins, 2017; Mora et al., 2017). The possibility of unintended consequences and adverse outcomes should also be considered and assessed as part of evaluations even when posited to have health benefits (e.g. including displacement as an unintended consequence of physical upgrading).

Finally, the definition and changing nature of interventions as they are implemented can pose a challenge for interpretation (Humphreys et al., 2016). Recipients of the intervention may be defined in advance based on household or geographic area, yet with variation in practice as to whether the intervention is received fully and exclusively by this group. These challenges can be met by adapting the evaluation to account for changing intervention plans and delivery, making pragmatic choices about which populations of recipients to assess for different parts of the evaluation, using multiple methods of evaluation concurrently (e.g. primary quantitative and qualitative data collection along with secondary data analyses), and building strong partnerships between evaluators and the organizations implementing urban interventions with opportunities to articulate and harmonize goals. Lastly, interpretation of reviews such as ours is challenging in the presence of publication bias. To limit this, the establishment of registries of evaluations could be very useful.

A number of the insights generated from our quality assessment of these urban upgrading evaluations have been echoed in general guidance for the evaluation of natural experiments (Craig et al., 2012). This affirms the persistent challenges of such evaluation, while resource and logistical constraints make such challenges particularly acute for evaluations within urban informal settlements.

While this review summarizes quantitative findings from physical environment upgrading interventions, other knowledge gaps remain and represent areas where further synthesis is needed. Slum upgrading can include social, legal, and economic interventions. Simultaneous interventions of these types could contribute to the observed findings of included evaluations. Systematic reviews of evaluations considering these interventions separately can create a more focused picture of how specific components of slum upgrading impact different health outcomes across settings. Also, while qualitative evidence was outside the scope of this review, such evidence could help contextualize variations observed across quantitative results.

## 5 Conclusion

The evaluation of health impacts of physical environment upgrading interventions remains an important need, yet a challenging task. Evidence of positive health effects, including those of housing on quality of life and communicable diseases, provides only limited insight as to which specific pathways contributed to health benefits of such interventions. Limitations noted for the quality of evidence and the diffuse nature of comprehensive interventions suggests a need to continue to invest in rigorously designed evaluations, including randomized controlled trials, with particular attention to validated and blinded outcome assessment, hypothesized causal mechanisms, and analytic approaches to appropriately account for issues that may arise related to confounding and missing data. In addition to posited health improvements, evaluators should consider collecting and analyzing data to capture unintended health consequences and environmental cobenefits. Understanding and isolating the effects of informal settlement upgrading and other place-based interventions can inform necessary policy decisions to address inadequate living conditions as rapid urban growth continues across the globe.

## Supplementary Material

Supplementary data to this article can be found online at https://doi.org/10.1016/j.socscimed.2020.113102.

Tables 1-5

## Figures and Tables

**Table 1:** Characteristics of informal settlement upgrading evaluations published between 1986 and 2018. A. Primary evaluations (in chronological order). B. Supporting evaluations (in chronological order).

A. Primary evaluations (in chronological order)														
		Turley’s review (up to 2012)					Curren review (2012-2018)							
		Taylor 1987	Soares 2005	Galiani 2007	Butala 2010	Gonzales-Navarro 2010	Cerdá 2012	Friche 2015	Ordóñez Barba 2013	Ordóñez Barba 2015	Gonzalez-Navarro 2016	Galliani 2017	Galliani 2018	McIntosh 2018
Physical interventions	Water and sanitation	**✓**	**✓**	**✓**	**✓**			**✓**	**✓**	**✓**				**✓**
	Improved housing		**✓**		**✓**			**✓**				**✓**	**✓**	
	Energy infrastructure				**✓**			**✓**	**✓**	**✓**				**✓**
	Environmental hazard	**✓**	**✓**		**✓**			**✓**						
	Waste management	**✓**	**✓**		**✓**			**✓**						
	Roads and transportation	**✓**	**✓**		**✓**	**✓**	**✓**	**✓**	**✓**	**✓**	**✓**			**✓**
Other interventions	Social environment							**✓**	**✓**	**✓**				**✓**
	Health education/behavior	**✓**			**✓**				**✓**	**✓**				**✓**
	Health/social services				**✓**			**✓**	**✓**	**✓**				**✓**
Health outcomes	Communicable diseases		**✓**	**✓**	**✓**	**✓**			**✓**		**✓**	**✓**		
	Nutritional deficiencies													
	Maternal and perinatal		**✓**											
	Injuries					**✓**								
	Non-communicable diseases													
	General physical health					**✓**								
	General mental health										**✓**			
	Quality of life					**✓**						**✓**	**✓**	
	Mortality							**✓**						
Other outcomes	Social capital									**✓**				**✓**
	Personal or neighborhood safety		**✓**			**✓**	**✓**		**✓**		**✓**	**✓**		**✓**
Location	Americas		**✓**	**✓**		**✓**	**✓**	**✓**	**✓**	**✓**	**✓**	**✓**	**✓**	**✓**
	Africa													
	Southeast Asia	**✓**			**✓**									
	Western Pacific													
	Other													
Study design	Randomized controlled trial					**✓**			**✓**	**✓**	**✓**	**✓**	**✓**	****✓****
	Controlled before-after	**✓**	**✓**	**✓**	**✓**		**✓**	**✓**						
	Uncontrolled before-after													
	Controlled post-intervention only													
Summary of evaluation characteristics, including intervention components, outcomes assessed, regional context, and study design for primary evaluations in the current review and the review by Turley et al. Check marks represent characteristics applicable to a given evaluation.														

B. Supporting evaluations (in chronological order)															
		Turley’s review (up to 2012)									Current review (2012-2018)				
		De Leon 1986	Moitra 1987	Milone 1993	Abelson 1996	Aiga 2002	Josh¡ 2002	Moraes 2004	Cattaneo 2009	Parikh in press	Parikh 2012	Shortt 2013	Simonelli 2013	Marais 2014	Parikh 2015
Physical interventions	Water and sanitation		**×**	**×**	**×**	**×**	**×**	**×**		**×**	**×**				**×**
	Improved housing	**×**			**×**		**×**		**×**			**×**	**×**	**×**	
	Energy infrastructure					**×**				**×**	**×**				**×**
	Environmental hazard	**×**	**×**	**×**	**×**		**×**	**×**		**×**	**×**				**×**
	Waste management	**×**		**×**			**×**			**×**	**×**				**×**
	Roads and transportation		**×**	**×**	**×**	**×**	**×**	**×**		**×**	**×**				**×**
Other intervention	Social environment	**×**	**×**		**×**										
	Health education/behavior	**×**			**×**										
	Health/social services		**×**		**×**		**×**								
Health outcomes	Communicable diseases			**×**		**×**		**×**	**×**			**×**		**×**	
	Nutritional deficiencies				**×**				**×**					**×**	
	Maternal and perinatal				**×**										
	Injuries											**×**			
	Non-communicable diseases				**×**				**×**					**×**	
	General physical health		**×**		**×**				**×**	**×**		**×**	**×**		**×**
	General mental health				**×**				**×**			**×**	**×**		
	Quality of life								**×**		**×**		**×**		
	Mortality													**×**	
Other outcomes	Social capital	**×**										**×**	**×**		
	Personal or neighborhood safety														
Location	Americas							**×**	**×**				**×**		
	Africa											**×**		**×**	
	Southeast Asia	**×**	**×**	**×**	**×**	**×**	**×**			**×**	**×**				**×**
	Western Pacific														
	Other														
Study design	Randomized controlled trial														
	Controlled before-after														
	Uncontrolled before-after			**×**	**×**		**×**						**×**		
	Controlled post-intervention only	**×**	**×**			**×**		**×**	**×**	**×**	**×**	**×**		**×**	**×**
Summary of evaluation characteristics, including intervention components, outcomes assessed, regional context, and study design for supporting evaluations in the current review and the review by Turley et al. Check marks represent characteristics applicable to a given evaluation.														

**Table 2:** Synthesis of intervention-specific impacts on health outcomes in primary and supporting evaluations. A. Primary evaluations. B. Supporting evaluations.

		Communicable diseases	Nutritional deficiencies	Maternal & perinatal	Injuries	Non-communicable diseases	General mental health	General physical health	Quality of life	Mortality	Personal or neighborhood safety	Social capital
A. Primary evaluations												
**Single Interventions**						**Housing Interventions**						
	TECHO	(+) (+)										
	Galiani et al., 2018								(+)(+)		NS	
	Galiani et al., 2017								(+)				
						**Transportation Interventions**						
	Metrocable										(+)(+)	
	Cerdá et al., 2012										
	Street asphalting	NS NS			NS			NS	NS		NS NS	
	Gonzalez-Navarro and Quintana-Domeque, 2016											
	Gonzalez Navarro 2010											
						**Sanitation Interventions**						
	Water expansion Galiani 2007	NS(+)										
**Comprehensive Interventions**	Programa HÃ!bitat	NS NS									(+)(+) NS	NS NS
	McIntosh et al., 2018											
	Ordóñez Barba and Ruiz Ochoa, 2015											
	Ordóñez Barba et al., 2013											
	Vila Viva									NT		
	Friche et al.,2015											
	Favela-Barrio Soares 2005	NS		NS	NS						NS	
	Slum upgrading Butala 2010	(+)										
Each outcome measured at each time point within the evaluations included in the current review and the review by Turley et al. is represented by a mark in the table. Reference: (+) = significant health-promoting direction of effects;												
NS= non-significant effect; NT = notsignificance tested.												
Note: The Jakarta Kampung Improvement Programstudied in Taylor (1987) was not included as it only involved the assessment of poverty and no other health outcome or any other outcome in the pathway to health.												

		Communicable diseases	Nutritional deficiencies	Maternal & perinatal	Injuries	Non-communicable diseases	General mental health	General physical health	Quality of life	Mortality	Personal or neighborhood safety	Social capital
B. Supporting evaluations												
**Single Interventions**	**Housing Interventions**											
	TECHO						(+)(+)(+)	(+) NS	(+)(+)			NS NS
	Simonelli et al., 2013						NS					
	South African Housing	NS NS	NS			NS				NS NS		
	Subsidy											
	Marais and Cloete, 2014											
	People’s Housing Program	NS			(+)		(+)	(+)NS				(+)
	Shortt and Hammett, 2013											
	Piso Firme	NS(+)	NS(+)			(+)	NS	NS	(+)			
	Cattaneo 2009											
**Comprehensive Interventions**	Slum Improvement Program							(+)(+)(+)	(+)			
	Parikh et al., 2015							NT				
	Parikh et al., 2012											
	Parikh in press											
	Barrio Escopa project											
	De Leon 1986											(+)
	Slum Improvement Program											
	Moitra 1987							NT				
	Small Towns Urban	NT										
	Development Project											
	Milone 1993											
	Slum Improvement project		NS NT	NS NT				NT NT				
	Abelson 1996											
	Zonal Improvement Program	(+)										
	Aiga 2002											
	Multicomponent physical	NT NT NT										
	upgrading											
	Moraes 2004											
Each outcome measured at each time point within the evaluations included in the current review and the review by Turley et al. is represented by a mark in the table. Reference: (+)= significant health-promoting direction of effects;												
NS = non-significant effect; NT = not significance tested.												
Note: The Slum Networking intervention measured by Joshi (2002) was not included as it only involved the assessment of poverty and no other health outcome or any other outcome in the pathway to health.												

**Table 3:** Recommendations for the future evaluations of physical environment urban and informal settlement upgrading interventions.

Domain for improvement	Recommendations
**STUDY DESIGN**	**Randomization** Random allocation may occur through natural experiments where recipients are selected randomly of a waiting list for an in-situ upgrading project. Example: Galiani et al., 2017, Gonzalez-Navarro and Quintana-Domeque, 2016. **Control groups** Select one or more control groups that are similar to the intervention group on key characteristics. Assess the balance of confounders between the groups at baseline. Group balance on key confounders can be accomplished in the study design phase through restriction and matching. Consider using techniques like propensity score matching to create more balanced groups if there are “significant differences. Example: Cerdá et al., 2012. **Multiple time points** Collecting data at multiple time points before and after intervention allows for flexibility in analysis and assessing short-term versus longer- term effects. Example: Galiani et al., 2018, Simonelli et al., 2013. Consider using panel data, or data collected from the same individuals over time. Repeated cross-sectional data on different individuals may also be used, such as data from repeated government surveys.
**METHODOLOGICAL APPROACH**	**Selecting appropriate health outcomes** Select outcome measures and determine the timing of their measurement based on the causal mechanisms hypothesized to operate. **Blinded outcome assessment** Consider using secondary data sources that are collected without knowledge of the research questions and, in some cases, without knowledge of the intervention status of individuals or communities. When primary data is collected, report in the text the extent to which participants were aware of research questions, and researchers/ assessors were aware of participants' intervention status. **Loss to follow-up** Loss to follow-up should be minimized by repeating data collection with the same measures for the same individuals at multiple time points. If loss to follow-up occurs, determine its magnitude and report whether loss to follow-up is balanced across group. Sensitivity analyses can help determine if loss to follow-up is a threat to the study's validity.
**ANALYTICAL APPROACH**	**Measures** Consider using measures of outcome and covariates that are already validated and reliable, based on previous studies and literature review. Pilot test any unique or ad-hoc data collection instruments in order to test validity and reliability. Use of untested measures should be accompanied by a strong theoretical basis for face validity. Example: Ordóñez Barba and Ruiz Ochoa, 2015. **Confounders** Determine potential confounders, mediators, and moderators in the study based on causal pathways using techniques like causal loop diagrams or directed acyclic graphs. Control for relevant confounders in order to assess the effects of the intervention. Domains of confounders to consider include: - individual socio-demographic characteristics (e.g. age, sex, race, income, education level) - place-based health determinants (e.g. community violence, walkability, air pollution, urbanicity) - pre-intervention assessment on outcomes of interest (e.g. baseline health status, baseline physical activity levels) Examples: Galiani et al., 2017, Cerdá et al., 2012. **Missing data** Clearly report the presence and extent of missing data. Include the reasons for missingness and the underlying pattern of missingness (i.e., missing completely at random, missing at random, not random missingness). Select ways to handle missing data that are appropriate for the type of missingness and characteristics of the data (e.g., imputation methods if missing is completely at random or otherwise complete case analysis). Specify in the methods section of the evaluation how missing data was handled.
