# Supplementary material for: Evaluating the health effects of place-based slum upgrading physical environment interventions: A systematic review (2012–2018)
Source: Soc Sci Med. Author manuscript; Available in PMC 2021 Aug 5. (PMC7611465; doi:10.1016/j.socscimed.2020.113102)
Supplement: Tables 1-5 [file EMS129602-supplement-Tables_1_5.docx]

## Supplementary Table 1. Search Strategy by Database

| **Database:** ProQuest  Applied Social Sciences Index & Abstracts (ASSIA), GeoRef (including in process), and Sociological Abstracts  All Source type, All language  Date range: Jan 1 2012-Dec 31 2018  **Date searched**: Jan 26 2019 | (ALL("public tap*" OR "tube well*" OR standpipe* OR "dug well" OR "septic tank*" OR toilet* OR "scal* up" OR water OR sanitation OR sewerage) OR ALL((waste OR garbage OR refuse) AND (collect* OR manag* OR service* OR system* OR dispos*)) OR ALL("storm drain*" OR electricity OR gas OR "power supply" OR "energy supply" OR utilities) OR ALL(subsidy OR subsidi* OR investment* OR partnership* OR microcredit OR credit OR "village bank*" OR microcredit* OR mortgage* OR microfinance OR loan OR loans) OR ALL(road* OR drainage OR "street light*" OR sidewalk OR pavement* OR infrastructure) OR ALL(improv* OR upgrad* OR transform* OR retransform* OR redevelop* OR renewal OR rehabilit* OR regenerat*) OR ALL("Communit* involv*" OR "participatory model*" OR "participat* communit*" OR "communit* manag*" OR "communit* organi*" OR "communit* group*") OR ALL(kip OR housing OR home OR homes)) AND (ALL("informal settlement*" OR "illegal settlement*" OR "squatter settlement*") OR ALL(shanty OR shanties OR shantyhouse* OR favela* OR kampung) OR ALL(slum OR slums)) **Results retrieved: 494** |
| --- | --- |
| **Database**: Ovid MEDLINE 1996-Jan Wk3 2019 & MEDLINE In-Process Jan 25 2019  Map Term to Subject Heading checked  Limits 2012-2018  **Date searched**: Jan 26 2019 | 1 poverty/ and (urban population/ or urban health/ or urbanization/)  2 Poverty Areas/  3 (slum or slums or shanty or shanties or shantyhouse* or barrio or barrios or favela* or kampung or tugurio* or ghetto or ghettos or bidon or bidons or bidonville*).ti,ab.  4 (informal shack* or irregular shack* or illegal shack* or shack dweller* or shackdweller or shack town* or skid row or urban blight or squatter camp* or shack settlement* or arrabal or asentamiento*or campamento* or cantegril* or comuna or comunas or Pueblos jovene* or barriada* or Poblacion callampa or tugurio or precario or chacarita or tent cit* or informal cit* or imijondolo*).ti,ab.  5 (bustee* or gecekondu* or chawls* or basti or masseque* or squatter cit* or katchi abadi* or dharavi or kibera or cite soleil or khayelitsha or orangi town).ti,ab.  6 (kartonsko naselje or habitat precaire or habitat spontane or quartier irregulier or asentamiento irregular or colonia popular or villa miseria or ciudad perdida or edina achouaia or mudal safi or pelli gewal).ti,ab.  7 (lahbach or elendsviertel or brarek or foundouks or tanake or aashwa or truschobi or taudis or morro or loteamento or comunidade or ahata or katra or watta or jhopadpatti or umjondolo or mabanda or kijiji or barraca or conventillos).ti,ab.  8 (cardboard cit* or tenement district* or tenement hous* or rundown neighborhood* or rundown neighbourhood* or rundown settlement* or immigrant camp$1 or immigrant settlement$ or migrant camp$1 or migrant settlement$ or refugee camp$1 or refugee settlement$1).ti,ab.  9 (public squalor or public squalor or squalid housing or squalid accommodation or human settlement development or urban poor).ti,ab.  10 ((informal adj2 shack*) or (informal adj2 settlement*) or (irregular adj2 settlement*) or (illegal adj2 settlement*) or (informal adj2 tenement*) or (irregular adj2 tenement*) or (squatter adj2 settlement*) or (squatter adj2 area*) or (illegal adj2 tenement*)).ti,ab.  11 or/1-10  12 ((secur* or land or propert) adj2 (tenur* or rights or titl*)).ti,ab.  13 (Communit* involv* or participatorymodel* or participat* communit* or communit*manag* or communit* organi* or communit* group*).ti,ab.  14 (kip or permanent hous* or formal hous* or hous* building or new hous*).ti,ab.  15 (improv* or upgrad* or transform* or retransform* or redevelop* or renewal or rehabilit* or regenerat*).ti,ab.  16 ((waste or garbage or refuse) adj2 (collect*OR service*OR manag* dispos*OR scheme* or remov*)).ti,ab.  17 (sewer system* or buil* road* or new road* or road* buil* or access road* or drainage system or underground sewerage or street light* or pave* road* or pave* footpath* or pave* sidewalk or pavement* or (infrastructure adj2 develop*)).ti,ab.  18 (rain water collection or piped water or public tap* or tube well* or standpipe* or protected spring or dug well or deliver* water or septic tank* or toilet* or safe water or borewell* or bore well* or drinking water).ti,ab.  19 ((water or sanitation or sewerage) adj2 (suppl* or prov* or expan* or access or service* or system$1)).ti,ab.  20 (subsid* or investment* or partnership* or microcredit or credit or village bank* or microcredit* or mortgage* or social fund* or microfinance or loan or loans or monetary support or monetary assis* or financ* support or financ* assist*).ti,ab.  21 ((storm adj2 drain*) or (environment* adj2 hazard*) or (mitigat* adj2 hazard*) or electricity or gas or power supply or energy supply or utilities).ti,ab.  22 (Housing strateg* or housing polic* or housing project* or housing program* or new* buil* or social housing* or SI project* or urban management or manpower or capacity or political accountability or scal* up or home or homes or dwelling* or floor or floors or flooring).ti,ab.  23 water purification/ or water supply/ or urban renewal/ or waste management/ or urban renewal/ or consumer participation/  24 (flood protect* or ground stabili* or slope stabili* or (mitigat* adj2 landslide) or (drain* adj2 stormwater*) or (drain* adj2 surface) or (drain* adj2 system*) or (drain* adj2 water*) or (sewage adj2 collect*) or (sewage adj2 dispos*) or (sewage adj2 manag*) or (sewage adj2 remov*) or (sewage adj2 service*) or (sewage adj2 system*) or (sewage adj2 treatment*) or (sewer* adj2 collect*) or (sewer* adj2 dispos*) or (sewer* adj2 expansion) or (sewer* adj2 manag*) or (sewer* adj2 prov*) or (sewer* adj2 remov*) or (sewer* adj2 service*) or (sewer* adj2 suppl*) or (sewer* adj2 system*) or (sewer* adj2 treatment*) or (trash adj2 collect*) or (trash adj2 dispos*) or (trash adj2 dispos*) or (trash adj2 manag*) or (trash adj2 remov*) or (trash adj2 service*) or (waste adj2 scheme*) or (manag* adj2 stormwater*) or (manag* adj2 surface water)).ti,ab.  25 ((electric adj2 cooker*) or (electric adj2 cookstove*) or (electric adj2 stove*) or (electric adj2 stoves) or (electric* adj2 provid*) or (electric* adj2 provis*) or (electric* adj2 suppl*) or (garbage adj2 service*) or (gas adj2 cooker*) or (gas adj2 cookstove*) or (gas adj2 stove*) or (gas adj2 stoves) or (improv* adj2 cooker*) or (improv* adj2 cookstove*) or (improv* adj2 stove*) or (improv* adj2 stoves) or (lpg adj2 cooker*) or (lpg adj2 cookstove*) or (lpg adj2 stove*) or (lpg adj2 stoves)).ti,ab.  26 or/12-25  27 11 and 26  28 limit 27 to yr="2012 - 2018"  **Results retrieved: 1,919** |
| **Database**: Web of Science Core Collection (Science Citation Index Expanded (SCI-EXPANDED) --1980-present  Social Sciences Citation Index (SSCI) --1980-present  Arts & Humanities Citation Index (A&HCI) --1980-present  Emerging Sources Citation Index (ESCI) --2015-present)  All languages, all document types  Years 2012-2018  **Date searched**: Jan 26 2019 | # 6  #5 OR #4  Indexes=SCI-EXPANDED, SSCI, A&HCI, ESCI Timespan=2012-2018  # 5  #3 AND #1  Indexes=SCI-EXPANDED, SSCI, A&HCI, ESCI Timespan=2012-2018  # 4  #3 AND #2  Indexes=SCI-EXPANDED, SSCI, A&HCI, ESCI Timespan=2012-2018  # 3  TS=((informal OR illegal OR informal) SAME (settlement*)) OR TS=(slum OR slums OR shanty OR shanties OR shantyhouse* OR barrio OR barrios OR favela* OR kampung)  Indexes=SCI-EXPANDED, SSCI, A&HCI, ESCI Timespan=2012-2018  # 2  TS=(road* OR drainage OR “street light*” OR sidewalk OR pavement* OR infrastructure) OR TS=(improv* OR upgrad* OR transform* OR retransform* OR redevelop* OR renewal OR rehabilit* OR regenerat*) OR TS=(“Communit* involv*” OR “participatory model*” OR “participat* communit*” OR “communit* manag*” OR “communit* organi*” OR “communit* group*”) OR TS=((informal OR illegal OR informal) SAME (settlement*))  Indexes=SCI-EXPANDED, SSCI, A&HCI, ESCI Timespan=2012-2018  # 1  TS=(kip or housing or home* OR “public tap*” OR “tube well*” OR standpipe* OR “dug well” OR “septic tank*” OR toilet* OR “scal* up”) OR TS=(water OR sanitation OR sewerage) OR TS=(waste or garbage or refuse) OR TS=(“storm drain*” OR electricity OR gas OR “power supply” OR “energy supply” OR utilities ) OR TS=(subsid* OR investment* OR partnership* OR microcredit OR credit OR village bank* OR microcredit* OR mortgage* OR social fund* OR microfinance OR loan OR loans)  Indexes=SCI-EXPANDED, SSCI, A&HCI, ESCI Timespan=2012-2018  **Results retrieved: 2,638** |
| Cochrane Public Health Group Specialized Register  **Database:** Chochrane Reviews – Review  Chochrane Review Group: Public Health Group  Dates: Publication Year 2012-2018  **Date searched**: Jan 26 2019 | slum or slums or squatters or barrio or barrios or favela or favelas or shanty  **Results retrieved: 3** |
| Cochrane Central Register of Controlled Trials (CENTRAL)  **Database**: Trials  Dates: Publication Year 2012-2018  **Date searched**: Jan 26 2019 | #1 (poverty) and (“urban population” or “urban health” or urbanization):ti,ab,kw  #2 “Poverty Areas”:ti,ab,kw  #3 (slum or slums or shanty or shanties or shantyhouse* or barrio or barrios or favela* or kampung or tugurio* or ghetto or ghettos  or bidon or bidons or bidonville*) :ti,ab,kw  #4 (“informal shack*” or “irregular shack*” or “illegal shack*” or “shack dweller*” or shackdweller or “shack town*” or “skid row”  or “urban blight squatter camp*” or “shack settlement*” or arrabal or asentamiento* or campamento* or cantegril* or comuna or  comunas or “Pueblos jovene*” or barriada* or “Poblacion callampa” or tugurio or precario or chacarita or “tent cit*” or “informal cit*”  or imijondolo*) :ti,ab,kw  #5 (bustee* or gecekondu* or chawls* or basti or masseque* or “squatter cit*” or “katchi abadi*” or dharavi or kibera or “cite soleil”  or khayelitsha or “orangi town”):ti,ab,kw  #6 (lahbach or elendsviertel or brarek or foundouks or tanake or aashwa or truschobi or taudis ormorro or loteamento or comunidade  or ahata or katra or watta or jhopadpatti or umjondolo or mabanda or kijiji or barraca or conventillos) :ti,ab,kw  #7 (“public squalor” or “public squalor” or “squalid housing” or “squalid accommodation” or “human settlement development” or  “urban poor”):ti,ab,kw  #8 ((informalNEAR/2 shack*) or (informalNEAR/2 settlement*) or (irregularNEAR/2 settlement*) or (illegalNEAR/2 settlement*)  or (informal NEAR/2 tenement*) or (irregular NEAR/2 tenement*) or (squatter NEAR/2 settlement*) or (squatter NEAR/2 area*) or  (illegal NEAR/2 tenement*)):ti,ab,kw  #9 (“kartonsko naselje” or “habitat precaire” or “habitat spontane” or “quartier irregulier” or “asentamiento irregular” or “colonia  popular” or “villa miseria” or “ciudad perdida” or “edina achouaia” or “mudal safi” or “pelli gewal”) :ti,ab,kw  #10 (“cardboard cit*” or “tenement district*” or “tenement hous*” or “rundown neighborhood*” or “rundown neighbourhood*” or  “rundown settlement*”) :ti,ab,kw  #11 (#1 OR #2 OR #3 OR #4 OR #5 OR #6 OR #7 OR #8 OR #9 OR #10)  #12 ((secur* or land or propert) NEAR/2 (tenur* or rights or titl*)):ti,ab,kw  #13 (“Communit* involv*” or “participatory model*” or “participat* communit*” or “communit* manag*” or “communit* organi*”  or “communit* group*”):ti,ab,kw  #14 (kip or “permanent hous*” or “formal hous*” or “hous* building” or “new hous*”):ti,ab,kw  #15 (improv* or upgrad* or transform* or retransform* or redevelop* or renewal or rehabilit* or regenerat*):ti,ab,kw  #16 ((waste or garbage or refuse) NEAR/2 (collect* OR service* OR “manag* dispos*” OR scheme* or remov*)):ti,ab,kw  #17 (“sewer system*” or “buil* road*” or “new road*” or “road* buil*” or “access road*” or “drainage system” or “underground  sewerage” or “street light*” or “pave* road*” or “pave* footpath*” or “pave* sidewalk” or pavement* or (infrastructure NEAR/2  develop*)):ti,ab,kw  18 (“rain water collection” or “piped water” or “public tap*” or “tube well*” or standpipe* or “protected spring” or “dug well” or  “deliver* water” or “septic tank*” or toilet* or “safe water” or borewell* or “bore well*” or “drinking water”):ti,ab,kw  #19 ((water or sanitation or sewerage) NEAR/2 (suppl* OR prov* or expan* OR access or service* OR system$1)):ti,ab,kw  #20 (subsid* or investment* or partnership* or microcredit or credit or “village bank*” or microcredit* or mortgage* or “social fund*”  or microfinance or loan or loans or “monetary support” or “monetary assis*” or “financ* support” or “financ* assist*”):ti,ab,kw  #21 ((storm NEAR/2 drain*) or (environment* NEAR/2 hazard*) or (mitigat* NEAR/2 hazard*) or electricity or gas or “power  supply” or “energy supply” or utilities):ti,ab,kw  #22 (“Housing strateg*” or “housing polic*” or “housing project*” or “housing program*” or “new* buil*” or “social housing*” or “SI  project*” or “urban management” or manpower or capacity or “political accountability” or “scal* up” or home or homes or dwelling*):  ti,ab,kw  #23 (“water purification”) or (“water supply”) or (“urban renewal”) or (“waste management”) or (“urban renewal”) or (“consumer  participation”):ti,ab,kw  #24 (#12 OR #13 OR #14 OR #15 OR #16 OR #17 OR #18 OR #19 OR #20 OR #21 OR #22 OR #23)  #25 (#11 AND #24)  **Results retrieved: 159** |
| EBSCO  **Database:** GreenFILE &  Avery Index to Architectural Periodicals  Date published: 2012-2018  Date searched: Jan 26 2019 | S15 S12 and S13 Limiters - Date Published: 20120101-20181231  S14 S12 and S13  S13 S1 or S2 or S3 or S4 or S5 or S6 or S7 or S8  S12 S9 or S10 or S11  S11 TI ( slum or slums ) OR SU ( slum or slums ) OR AB ( slum or slums ) OR KW ( slum or slums )  S10 TX (shanty OR shanties OR shantyhouse* OR favela* OR kampung)  S9 TX (“informal settlement*” or “illegal settlement*” or “squatter settlement*”)  S8 TX (kip or housing or home or homes)  S7 TX (“Communit* involv*” OR “participatory model*” OR “participat* communit*” OR “communit* manag*” OR “communit* organi*” OR “communit* group*”)  S6 TX (improv* OR upgrad* OR transform* OR retransform* OR redevelop* OR renewal OR rehabilit* OR regenerat*)  S5 TX (road* OR drainage OR “street light*” OR sidewalk OR pavement* OR infrastructure)  S4 TX (subsidy OR subsidi* OR investment* OR partnership* OR microcredit OR credit OR “village bank*” OR microcredit* OR mortgage* OR microfinance OR loan OR loans)  S3 TX (“storm drain*” OR electricity OR gas OR “power supply” OR “energy supply” OR utilities )  S2 TX((waste or garbage or refuse) AND (collect* or manag* or service* or system* or dispos*))  S1 TX (“public tap*” OR “tube well*” OR standpipe* OR “dug well” OR “septic tank*” OR toilet* OR “scal* up” or water OR sanitation OR sewerage)  **Results retrieved: 307** |
| Ei Engineering Village  **Database**: Compendex  Years: 2012-2018  Expert Search  **Date searched**: Jan 26 2019 | #12 #11 AND #10  #11 slum OR slums OR kampung* OR favela* OR bidon* OR tugurio* OR {informal settlement*} OR {illegal settlement*} OR {squatter settlement*} OR {shack settlement*} OR {shack dwell*}  ### #11 restricted to years 2012-2018###  #10 #3 OR #4 OR #5 OR #6 OR #7 OR #8 OR #9  #9 kip OR housing OR home OR homes  #8 {Communit* involv*} OR {participatory model*} OR {participat* communit*} OR {communit* manag*} OR {communit* organi*} OR {communit* group*}  #7 improv* OR upgrad* OR transform* OR retransform* OR redevelop* OR renewal OR rehabilit* OR regenerat*  #6 road* OR drainage OR {street light*} OR sidewalk OR pavement* OR infrastructure OR subsidy OR subsidi* OR investment* OR partnership*  #5 microcredit OR credit OR {village bank*} OR microcredit* OR mortgage* OR microfinance OR loan OR loans  #4 {storm drain*} OR electricity OR gas OR {power supply} OR {energy supply} OR utilities OR {public tap*} OR {tube well*} OR standpipe* OR {dug well} OR {septic tank*} OR toilet* OR {scal* up} OR water OR sanitation OR sewerage  #3 #1 AND #2  #2 (waste OR garbage OR refuse)  #1 (collect* OR manag* OR service* OR system* OR dispos*)  **Results retrieved: 381** |

## Supplementary Table 2. Grey literature and development stakeholder sites searched

| **Name of the organization** | **Type of web-based information consulted** |
| --- | --- |
| Asian Development Bank | Evaluation Documents: 04/01/2012-10/05/2018 |
| Care International | Annual reports |
| Centers for Disease Control and Prevention | Home page search |
| Cities Alliance | Our knowledge |
| Comic Relief | Home page search |
| International Council for Research and Innovation in Building and Construction (CIBD) | Home page search |
| The Abdul Latif Jameel Poverty Action Lab (J-PAL) | Evaluations |
| Office of Evaluation and Oversight (OVE) of the Inter-American Development Bank | Evaluations: Projects type |
| Oxfam International | Evaluations and Publications: Research |
| Red Cross | Home page search |
| Slum Dwellers International | Home page search |
| The Society for the Promotion of Area Resource Centres (SPARC) | Home page search |
| UK Department for International Development (DFID) | Home page search |
| UN-HABITAT | Centralized Evaluations  Decentralized Evaluations  Other Entity Evaluations |
| US Agency for International Development (USAID), including its Environmental Health Project (EHP) | Home page search |
| Water Aid | Home page search |
| World Bank | Knowledge repository: Annual reports and independent evaluations |
| World Health Organization | Iris WHO digital library |

## Supplementary Table 3. Effective Public Health Practice Project Quality Assessment Tool for Quantitative Studies’ Quality Assessment Criteria (Adapted for natural experiments)

| **Domain** | **Quality Rating Criteria** |
| --- | --- |
| **SELECTION BIAS** | *Strong*: The selected individuals are very likely to be representative of the target population and there is greater than 80% participation |
|  | *Moderate*: The selected individuals are at least somewhat likely to be representative of the target population and there is 60 - 79% participation or the percentage of individuals participating cannot be determined. |
|  | *Weak*: The selected individuals are not likely to be representative of the target population OR there is less than 60% participation OR selection is not described and the level of participation is not described. |
| **STUDY DESIGN** | *Strong*: will be assigned to those articles that described RCTs and CCTs |
|  | *Moderate*: will be assigned to those that described a cohort analytic study (controlled before-after), a case control study, a cohort design, or an interrupted time series |
|  | *Weak*: will be assigned to those that used any other method or did not state the method used |
| **INTERVENTION INTEGRITY** | *Strong*: 80% or more of participants receive the allocated intervention as assigned and the allocated intervention is described as being consistent or administered with negligible variation and the control group did not clearly receive intervention contamination that biases the effect estimate |
|  | *Moderate*: 60-79% of participants received the intervention as assigned and the allocated intervention is described as consistent or administered with negligible variation and the control group did not clearly receive intervention contamination that biases the effect estimate OR 80% or more of participants receive the allocated intervention as assigned, but consistent allocation of the intervention is NOT described or measured and the control group did not clearly receive intervention contamination that biases the effect estimate |
|  | *Weak*: Less than 60% of participants received the intervention as assigned or the number who received the intended intervention is not described regardless of consistency or contamination OR 60-79% of participants received the intervention as assigned, but consistent allocation of the intervention is NOT described or measured and the control group received intervention contamination that biases the effect estimate |
| **BLINDED OUTCOME ASSESSMENT** | *Strong*: The outcome assessor is blinded to the intervention status of participants and the study participants are blinded to the research question |
|  | *Moderate*: The outcome assessor is blinded to the intervention status of participants but blinding of participants is not described OR the study participants are blinded to the research question but blinding of outcome assessors is not described |
|  | *Weak*: The outcome assessor is aware of the intervention status of participants and the study participants are aware of the research question OR blinding of both assessors and participants is not described |
| **DATA COLLECTION METHODS** | *Strong*: In general, the data collection tools have been shown to be valid and the data collection tools have been shown to be reliable |
|  | *Moderate*: In general, the data collection tools have been shown to be valid but the data collection tools have not been shown to be reliable or reliability is not described |
|  | *Weak*: In general, the data collection tools have not been shown to be valid regardless of their reliability |
| **WITHDRAWALS AND DROPOUTS** | *Strong*: will be assigned when the follow-up rate is 80% or greater |
|  | *Moderate*: will be assigned when the follow-up rate is 60 – 79% |
|  | *Weak*: will be assigned when longitudinal assessment of the same participants was not attempted in a study with repeated data collection OR a follow-up rate is less than 60% OR if the withdrawals and drop-outs were not described OR drop-outs are not balanced across groups and are likely to have an effect on the outcome |
|  | *Not Applicable*: Withdrawals and drop-outs are not applicable because the study design does not include longitudinal follow up or repeated measures |
| **CONFOUNDERS** | *Strong*: there are no important differences between intervention and control groups before intervention OR the study controlled for at least 75% of relevant confounders |
|  | *Moderate*: the study controlled for 50 – 74% of relevant confounders |
|  | *Weak*: less than 50% of relevant confounders were controlled OR control of confounders was not described |
| **ANALYSES** | *Strong*: The measures of association are appropriate, and the statistical methods are appropriate, and the analysis is performed by intention to intervene, and missing data is handled properly |
|  | *Moderate*: The measures of association are appropriate, and the statistical methods are appropriate, but the analysis is performed by intervention received or is not described, and missing data is handled properly |
|  | *Weak*: The measures of association are not appropriate or not described, OR the statistical methods are not appropriate, or not described OR missing data is not handled properly or not described – regardless of intention to treat analysis |

## Supplementary Table 4. Detailed summary of primary and supporting evaluations published between 2012 and 2018 A. Primary evaluations

| **Author-Year** | **Study Design** | **Country of Intervention** | **Unit of analysis (UA) & sample size (SZ)** | **Main intervention assessed** | **Control variables** | **Main health outcomes** | **Findings** |
| --- | --- | --- | --- | --- | --- | --- | --- |
| Galiani 2017 | Randomized controlled trial^1^ | El Salvador  Mexico  Uruguay | UA: Households clustered in informal settlements.  SZ: 2373 households (1356 in treated group and 1017 in control group) in 74 settlements.  Sample size by country: El Salvador (421 in treated group and 277 in control group clustered in 23 settlements), Mexico (457 in treated group and 439 in control group clustered in 39 settlements), and Uruguay (478 in treated group and 301 in control group clustered in 12 settlements). | Type: Single housing intervention  Name: TECHO Description: provision of housing units of 18 m^2^ made of pre-fabricated, insulated pinewood or aluminum panels with no service connection | Individuals socio-demographic characteristics Socio-economic characteristics of countries | Quality of life  Child respiratory episodes in the last 4 weeks  Child diarrhea episodes in the last 4 weeks  Perception of security | +  +  +  + El Salvador/ NS México & Uruguay |
| Galiani 2018 | Randomized controlled trial | El Salvador  Mexico  Uruguay | UA: Households clustered in informal settlements  SZ:2210 households (927 in Phase I and 1283 in Phase II) clustered in 74 settlements.  Sample size by countries: El Salvador (288 in Phase I; 368 in Phase II clustered in 23 settlements), Mexico (286 in Phase I; 540 in Phase II clustered in 39 settlements), and Uruguay (353 in Phase I; 375 in Phase II clustered in 12 settlements). | Type: Single housing intervention  Name: TECHO | Individuals socio- demographic characteristics Socio-economic characteristics of settlements and countries | Quality of life | + but at a diminishing rate over time |
| Gonzalez Navarro 2016 | Randomized controlled trial | México | UA: Households clustered in street blocks  SZ: 1083 households in 56 street blocks (28 treated and 28 control street blocks).  Number of households intervened by intervention/control status is not described. Authors sampled intervened group at a rate of 70% and control group at 50%. | Type: Single transportation intervention  Name: N/A  Description: First-time asphalting of residential nonarterial streets. No other physical improvement was made in the area. | Individual demographic characteristics Household socio- economic characteristics | Sick previous month  Fungus/parasite skin infections  Feel safe walking at night | NS  NS  NS |
| McIntosh 2018 | Randomized controlled trial | México | UA: households clustered in polygons^3^ clustered in municipalities  SZ: 342 polygons (166 polygons in the intervention group and 176 polygons in the control group) clustered in 33 municipalities).  4567 households in the intervention group and 5135 in the control group were interviewed at baseline, while 4573 and 5145 were interviewed after 2 years of follow-up, respectively. | Type: Comprehensive intervention  Name: Programa Hábitat  Description: a federal comprehensive urban infrastructure program that involves public infrastructure (street paving, sidewalks construction, service provision), the creation of community  development centers, job training, and health and nutrition training for young  mothers. | Socio-demographic characteristics of polygons and municipalities | Social capital  Physical assault  Teen behavior | NS  +  + |
| Ordóñez Barba 2013 | Randomized controlled trial | México | UA: Households clustered in polygons clustered in municipalities.  SZ: 342 polygons (166 polygons in the intervention group and 176 in the control group) clustered in 33 municipalities. 4567 households in the intervention group and 5135 in the control group were interviewed at baseline, while 4573 and 5145 were interviewed after 2 years of follow- up, respectively. | Type: Comprehensive intervention  Name: Programa Hábitat | Socio-demographic characteristics of polygons and municipalities | Safety limits activities  Diarrhea episodes in last 3 months  Skin infection episodes in last 3 months | NS  NS  NS |
| Ordóñez Barba 2015 | Randomized controlled trial | México | UA: Households clustered in polygons clustered in municipalities.  SZ: 342 polygons (166 in the intervention group and 176 in the control group) clustered in 33 municipalities.  4567 households in the intervention group and 5135 in the control group were interviewed at baseline, while 4573 and 5145 were interviewed after 2 years of follow- up, respectively. | Type: Comprehensive intervention  Name: Programa Hábitat | Socio-demographic characteristics of households and polygons | Social capital | NS |
| Cerdá 2012 | Controlled before-after^2^ | Colombia | UA: respondents clustered in neighborhoods  SZ: 466 respondents in 28 neighborhoods (255 participants from 25 serviced neighborhoods and 241 in 23 not-serviced neighborhoods interviewed at baseline and after 5 years) | Type: Single transportation intervention  Name: Metrocable  Description: cable-propelled transit system. The line (K) opened in 2004, connecting the city center to the Santo Domingo neighborhood in the  periphery, with 4 stops, covering a distance of 2,072 m, and reaching an elevation of 399 m. | Individuals socio-economic and demographic characteristics Socio-economic characteristics of neighborhoods | Perceived violence  Annual neighborhood homicide rate | +  + |
| Friche 2015 | Controlled before-after | Brazil | UA: informal settlements and formal city  SZ: 1 intervened informal settlement and 1 not- intervened informal settlement selected by convenience, the formal city (not integrated by informal settlements) was considered as a whole. Number of people living in each area was not reported. | Type: Comprehensive intervention  Name: PAC-Vila Viva  Description: urban redevelopment of slums involving implementation and improvement of the roadway system, water supply and sewage networks, drainage, slope stabilization, housing improvements, removals and resettlements, land tenure regularization including titling, and promotion of the communities’ socioeconomic development | Age-adjustment | Cause-specific mortality rate (morality due to infectious diseases, non- communicable diseases, and external causes) | NT |
| Findings: “+” = significant health-promoting direction of effects; “NS” = non-significant effect; “NT” = not significance tested.  ^1^Randomized controlled trial: Individual participants are randomly assigned to an intervention or control group, and data collection on outcomes of interest occurring both before and after intervention receipt. ^2^Controlled before-after: Participants are non-randomly assigned to an intervention or control group by investigator allocation or self-selection, and data collection on outcomes of interest occur both before and after intervention receipt.  ^3^A polygon is an explicit geographic perimeter outlining a neighborhood consisting of settled households in marginalized urban areas with concentrations of asset poverty greater than 50 percent, located in cities of 15,000 inhabitants or more, with a deficit of infrastructure and urban services, and with at least 80 percent of the lots having no active conflict over property rights. A Hábitat polygon is smaller than a locality and is a designation not used by other layers of government. | | | | | | | |

1. Supporting evaluations

| **Author-Year** | **Study Design** | **Country of Intervention** | **Unit of analysis & sample size** | **Main intervention assessed** | **Control variables** | **Main health outcomes** | **Findings** |
| --- | --- | --- | --- | --- | --- | --- | --- |
| Simonelli 2013 | Uncontrolled before-after^1^ | Argentina | UA: Individuals clustered in households.  SZ: 150 individuals interviewed at baseline, 77 at 1st month follow- up, 30 at 6th month follow-up). Number of households not specified. | Type: Single housing intervention  Name: TECHO | Individuals socio-demographic characteristics | Quality of life  Physical health  Psychological well-being  Social relationships  Sleep quality | + at 1 month/ + at 6 months  + at 1 month/ NS at 6 months  + at 1 month/NS at 6 months  NS at 1 month/ NS at 6 months  + at 1 month/ + at 6 months |
| Marais 2014 | Controlled post-intervention^2^ | South Africa | UA: individuals clustered in households  SZ: 26,000 individuals (16,500 adults and 9,500 children) clustered in 7,300 households. | Type: Single housing intervention  Name: South African housing subsidy program  Description: governmental housing subsidy for low-income residents to construct or improve housing units | Individuals socio-demographic and characteristics | Child nutrition  Child mortality  Infant mortality  Adult self-reported infectious disease symptoms  Adult TB diagnosis  Adult asthma diagnosis | NS  NS  NS  NS  NS  NS |
| Shortt 2013 | Controlled post- intervention | South Africa | UA: households  SZ: 201 households  (92 ‘upgraded’ and 109 non-intervened). | Type: Single housing intervention  Name: People’s Housing Process (PHP)  Description: governmental housing subsidy for low-income residents in which construction is driven by the local community | Individuals socio-demographic characteristics | Reporting illness or injury  Mental illness  Self-rated health  Skin disorders  Community belonging | +  +  NS  NS  + |
| Parikh 2012 | Controlled post- intervention | India | UA: households clustered in informal settlements selected by convenience  SZ: 500 households in 5 informal settlements (300 households in 3 intervened informal settlements and 200 households in 2 control informal settlements). | Type: Comprehensive intervention  Name: Slum Networking  Description: in-situ slum upgrading program, that involves the provision of integrated household-level infrastructure in the form of water, sanitation, roads, electricity, rainwater drainage | Individuals socio-demographic characteristics  Socio-economic characteristics of settlements | Quality of life | + |
| Parikh 2015 | Controlled post- intervention | India | UA: households clustered in informal settlements selected by convenience  SZ: 500 households in 5 informal settlements (300 households in 3 intervened informal settlements and 200 households in 2 control informal settlements). | Type: Comprehensive intervention  Name: Slum Networking | Individuals socio- demographic characteristics  Socio-economic characteristics of settlements | Per-capita disease rate (annual disease incidence within each household divided by the family size)  Monthly medical expenditure  Monthly work days lost to illness | +  +  + |
| Findings: “+” = significant health-promoting direction of effects; “NS” = non-significant effect; “NT” = not significance tested.  ^1^Uncontrolled before-after: Participants are non-randomly assigned to an intervention group only through investigator allocation or self-selection, and data collection on outcomes of interest occur both before and after intervention receipt. ^2^Controlled post-intervention: Participants are non-randomly assigned to an intervention or control group by investigator allocation or self-selection, and data collection on outcomes of interest occur only after intervention receipt. | | | | | | | |

## Supplementary Table 5. Results from quality assessment of each domain in each evaluation published between 2012 and 2018

|  | **Participant selection** | **Study design** | **Intervention integrity & intensity** | **Blinded outcome assessment** | **Data collection** | **Loss to follow-up** | **Confounding** | **Analysis** |
| --- | --- | --- | --- | --- | --- | --- | --- | --- |
| **Galiani 2017** | 2 | 1 | 1 | 2 | 3 | 1 | 1 | 3 |
| **Galiani 2018** | 2 | 1 | 1 | 2 | 1 | 1 | 1 | 3 |
| **Gonzalez Navarro 2016** | 1 | 1 | 2 | 2 | 3 | 1 | 1 | 3 |
| **MacIntosh 2018** | 1 | 1 | 2 | 3 | 3 | 3 | 1 | 3 |
| **Ordóñez Barba 2013** | 2 | 1 | 2 | 2 | 3 | 2 | 3 | 2 |
| **Ordóñez Barba 2015** | 2 | 1 | 2 | 2 | 3 | 3 | 3 | 2 |
| **Cerda 2012** | 2 | 2 | 3 | 3 | 2 | 2 | 1 | 3 |
| **Friche 2015** | 3 | 2 | 3 | 2 | 2 | NA | 3 | 3 |
| **Simonelli 2013** | 2 | 2 | 2 | 3 | 2 | 3 | 3 | 3 |
| **Marais 2014** | 2 | 3 | 2 | 2 | 3 | NA | 3 | 3 |
| **Shortt 2013** | 3 | 3 | 2 | 3 | 3 | NA | 3 | 3 |
| **Parikh 2012** | 3 | 3 | 2 | 3 | 2 | NA | 3 | 3 |
| **Parikh 2015** | 2 | 3 | 2 | 3 | 3 | NA | 3 | 2 |
| Note: 1 = strong, 2 = moderate, 3 = weak, NA = not applicable | | | | | | | | |
